# Intervention changes acoustic peak frequency and mesolimbic neurochemistry in the *Pink1-/-* rat model of Parkinson disease

**DOI:** 10.1371/journal.pone.0220734

**Published:** 2019-08-02

**Authors:** Sharon A. Stevenson, Michelle R. Ciucci, Cynthia A. Kelm-Nelson

**Affiliations:** Department of Surgery, Division of Otolaryngology, University of Wisconsin-Madison, Madison, Wisconsin, United States of America; Northeastern Ohio Medical University, UNITED STATES

## Abstract

The neural mechanisms underlying behavioral therapy for vocal acoustic deficits in patients with Parkinson disease is unknown. A primary hypothesis is that voice therapy may modulate mesolimbic brainstem regions, including the ventral tegmental area (VTA). The VTA is implicated in ultrasonic call peak frequency, involved in rewarding behaviors, and impacted by Parkinsonism. We tested the hypothesis that chronic (daily) behavioral vocal exercise of male *Pink1*-/- rats would alter ultrasonic vocalization acoustics and mesolimbic neurochemistry (catecholamines, GABA, mu-opioid receptor) compared to three different controls: sham-exercised *Pink1*-/-, unexercised *Pink1*-/-, and unexercised wildtype (WT) rats. A sub-hypothesis is that sham-exercise rats may exhibit changes to VTA neurochemistry as a result of a type or rewarding intervention. Results demonstrate that average bandwidth (frequency range) of ultrasonic vocalizations did not differ between WT, *Pink1*-/- no exercise vs. sham and vocal-exercise rats. However, average peak frequency is significantly reduced in vocal-exercised *Pink1*-/- rats compared to *Pink1*-/- no exercise, and WT groups. Unexpectedly, there were no significant acoustic differences between the vocal- and sham-exercised groups. There were no differences in catecholamine protein concentrations or tyrosine hydroxylase mRNA expression in the VTA between any of the groups. However, there was significant upregulation of all GABA-related genes in both vocal- and sham-exercised *Pink1*-/- rats (*Gad1*, *Gad2*, *Gls*, *Glul*); this finding was confirmed with follow up quantitative Western blotting for GAD. Additionally, there were differential results for mu-opioid receptor quantification in the VTA: vocal-exercised *Pink1*-/- rats showed increased mRNA expression for mu-opioid receptors whereas Western blotting indicated decreased protein levels in all *Pink1*-/- rats compared to WT controls suggesting the possible onset of pathology in this model. These data demonstrate modulatory effects of a rewarding behavioral paradigm on ultrasonic vocalization peak frequency. The results suggest that neuromodulators such as GABA and opioid activity, as well as the rewarding aspects of therapy may play a key role in shaping vocal treatments.

## Introduction

Parkinson disease (PD) is a complex neurodegenerative disease that affects multiple sensorimotor systems [1], including significant impairment of vocal communication in the early, prodromal stages of the disease [2–4]. Dopamine replacement therapies aimed at the hallmark pathology of nigrostriatal dopamine loss improves many of the limb-motor, but not vocal-motor deficits [5–9]. This suggests that other mechanisms or neurotransmitters may be influencing early-onset vocalization deficits. PD vocal dysfunction is responsive to behavioral therapies [10–13] such as intensive exercise-based interventions, and benefits from social environment and enrichment [14–19]. As such, rewarding aspects of intervention may drive therapeutic success and contribute to modulation neural structures involved in vocal production and reward. Defining the mechanisms underlying why such interventions improve vocal communication may lead to more effective and targeted treatments for PD-related voice and communication deficits [9].

Neuronal regions that may be modulated by vocal exercise interventions include extra-striatal networks, such as reward and motivation pathways in the ascending mesolimbic system. There is significant anatomical heterogeneity within this system. The midbrain ventral tegmental area (VTA) is necessary for brainstem-mediated motor behaviors including vocalizations, and is significantly compromised in late-stage PD [20–22]. The VTA is primarily composed of both dopamine and gamma-aminobutyric acid (GABA) neurons where GABA neurons co-exist, innervate, and modulate dopamine release [23, 24]. Additionally, a subset of local GABA interneurons is mediated via endogenous opioid neuropeptides and their respective mu-opioid receptor. Opioids have been shown to remove GABA inhibition and increase dopamine cell activity via the hyperpolarization of this network [25], thus, influencing downstream projection sites including the nucleus accumbens, basolateral amygdala, and prefrontal cortex. For example, post-mortem analyses of the VTA in individuals with PD show decreases in dopamine markers [22, 26]. Additionally, GABA downregulation, within and outside of the central nervous system, is a common factor in PD (reviewed in [27]). These data suggest that the VTA is susceptible to PD-related pathology and may significantly contribute to vocal deficits; thus, the VTA is a good candidate for examining the neural modulation of vocalizations with behavioral interventions that are inherently rewarding.

Rodent models are commonly used to study PD-related sensorimotor behaviors including vocalizations. Rats use 50-kilohertz (kHz) frequency-modulated ultrasonic vocalizations (USVs) to communicate in a variety of social situations, such as socio-sexual mating paradigms, and are used to investigate how PD-related pathology influences these vocalizations [28–42]. Social USVs are affiliative, appetitive, and transmit emotional states to conspecifics [30, 43–45]. Moreover, these vocalizations are directly associated with motivation and reward stemming from the mesolimbic system including the VTA. For example, anticipation of play increases the rate of 50-kHz vocalizations in juvenile rats [46], as does the anticipation of rewarding stimuli [47]. The release of VTA dopamine has been associated with modulating the frequency (bandwidth and peak energy frequency) of 50-kHz vocalizations. For example, drug microinjections into the mesolimbic dopamine system elicit 50-kHz vocalizations [48–50], and specific electrical stimulation of the VTA increases 50-kHz calls. Congruently, electrolytic lesions, dopamine antagonists, and 6-hydroxydopamine (6-OHDA) lesions reduce 50-kHz vocalizations, specifically decreasing the bandwidth frequency and peak frequency [37, 50, 51]. Thus, the mesolimbic VTA is well positioned to modulate the frequency of rat USVs.

Vocal communication deficits in rats that parallel human dysfunction have been quantified in multiple models of PD including the *Pink1*-/- rat, a genetic model of PD [37, 38]; however, the role of the mesolimbic VTA and the relationship to USV peak acoustic frequency has not been examined. *Pink1-/-* rats exhibit early, progressive 50-kHz USV deficits [52] that compromise functional communication by 8 months (mo) of age [53]. In addition, *Pink1*-/- rats exhibit early-onset brain pathology including abnormal alpha-synuclein protein aggregations in the periaqueductal gray and nucleus ambiguus [52, 54], show early metabolic and mitochondrial degeneration in the cortex and striatum [55], and have reductions in locus coeruleus norepinephrine [52, 56]. Similar to humans with PD, peripheral levodopa administration does not significantly improve vocalization deficits in *Pink1*-/- rats [57]. Additionally, Kelm-Nelson 2016 suggests that daily social contact (male-female interaction) and experimental handling improves acoustic parameters compared to rats that did not receive these procedures [58]. To date, the modulation of neurochemistry within the mesolimbic VTA in the *Pink1*-/- model has not been measured.

To address the greater role of the VTA in the vocalizing *Pink1*-/- rat, we analyzed male USVs and evaluated VTA catecholamine protein concentrations (dopamine and norepinephrine) with *Th* mRNA expression, GABAergic mRNA expression (*Gad1*, *Gad2*, *Gls*, *Glul*) and GAD protein as well as mu-opioid receptor mRNA and protein expression in: three control conditions (1) normal wildtype (WT) rats, (2) control (non-exercised) *Pink1*-/- rats (3) sham-exercised *Pink1*-/- rats, and compared them to (4) vocal-exercised *Pink1*-/- rats. We hypothesized that *Pink1*-/- rats that were vocal-exercised would show improved vocal frequency (bandwidth and peak frequency) as well as different catecholamine protein and GABA and mu-opioid receptor expression compared to control groups. A sub-hypothesis tested in this study was that sham-exercise rats may exhibit changes to VTA neurochemistry as a result of a type of rewarding intervention and will have similar expression profiles as compared to the vocal-exercised animals. These data are important in testing the overarching hypothesis that social, vocalization-related therapies may have positive effects on the mesolimbic neuromodulatory system and vocalization behavior specific to PD.

## Materials and methods

### Animals and habituation

A total of 33 rats, aged 8 mo at testing, were used in the study (**Table 1**). Rats were randomized into four experimental groups (further defined below): WT non-exercise controls, control (non-exercised) *Pink1*-/-, sham-exercised *Pink1*-/-, and vocal-exercised *Pink1*-/- (SAGE Research Labs, Boyertown, PA, USA [59]). All sham- and vocal-exercised *Pink1*-/- rats were part of a previously described behavioral experiment [58]; however, this study is the first to describe brain neurochemistry with the added *Pink1*-/- and WT control rats (all non-exercised) [54, 58].

**Table 1:** Experimental sample size and groups.

Experiment	Group & Final Sample Size
USV	WT = 12 Control *Pink1*-/- = 7 Sham-Exercised *Pink1*-/- = 6 Vocal-Exercised *Pink1*-/- = 6
ELISA	WT = 13 Control *Pink1*-/- = 6 Sham-Exercised *Pink1*-/- = 6 Vocal-Exercised *Pink1*-/- = 5
RT qPCR	WT = 7 Control *Pink1*-/- = 7 Sham-Exercised *Pink1*-/- = 4 Vocal-Exercised *Pink1*-/- = 4
Western Blot	WT = 4 Control *Pink1*-/- = 4 Sham- & Vocal- Exercised *Pink1*-/- = 4

Sample sizes in final analysis. Abbreviations: WT = wildtype.

All rats were housed in groups of two (within treatment groups) in standard polycarbonate cages (290 mm x 533 mm x 210 mm) with sawdust bedding on a reversed 12:12 hour light: dark cycle. A subset of estrous females (n = 8) from the animal colony were used to elicit USVs and were not used in the statistical analysis. All acclimation and testing occurred during the dark period of the cycle under red light illumination. Food and water were available *ad libitum*. All procedures were approved by the University of Wisconsin-Madison Animal Care and Use Committee (IACUC) and were conducted in accordance with the National Institutes of Health Guide for the Care and Use of Laboratory Animals [60].

### Daily vocal exercise and sham-exercise

Kelm-Nelson, Yang, and Ciucci (2016) published the initial behavior for the sham- and vocal-exercised rats presented here (referred to as sham-exercised *Pink1*-/- and vocal-exercised *Pink1*-/-). To briefly review behavioral methods, the vocal-exercised *Pink1*-/- rats each received daily (5 days per week) vocal exercise or ‘training’ for a total of 6 mo (2 mo until 8 mo of age). Each night, rats were water restricted. During the vocalization training, each rat was separated from his cage mate (cage mate was placed in a holding cage), and the home cage with the testing rat was placed underneath the ultrasonic microphone (see below). The experimental rat could mount (up to two times) or spend 5 minutes with the female stimulus rat, which-ever came first. After the female was removed from the cage, the test rat received a water reward (2 sec of water) after producing a USV on a variable ratio 5 schedule. Each rat was rewarded 20 times per session each day. Access to water was then restored for three hours. This experimental paradigm has been used in the lab to reliably elicit calls from male rats [39, 58, 61, 62]. Sham-exercised rats received identical treatment; however, instead of receiving a water reward for producing a USV, the rat received a water reward for going to the front right side of the cage. At the 8-mo timepoint, the statistical analysis of vocalizations within the *Pink1*-/- vocal exercise group were homogenous with little variation between rats *(e*.*g*. no outliers, all rats showed similar vocalization profiles). Additionally, all rats within this group exhibited a variety of call types and had similar intensities on the final days of the vocal exercise training. All WT and control *Pink1*-/- rats, were pair-housed but did not receive the previously described experimental protocol.

### Ultrasonic vocalizations

For this study, all USVs analyzed were elicited at 8 mo of age on the final testing day. USVs were recorded and analyzed as described in previous work [52, 58]. Briefly, an ultrasonic microphone was attached to a panel in the top center of a 10 cm x 10 cm x 12 cm sound-isolated Plexiglas chamber. The microphone, which has a flat frequency response up to 150-kHz and a frequency response range of 10-180-kHz, was used for recording frequency-modulated 50-kHz USVs (CM16, Avisoft, Germany). Recording parameters were set to a 16-bit depth and a sampling rate of 250-kHz.The subject rat was placed in the home cage within this chamber. The sexually receptive female stimulus rat was placed in the cage for two minutes. After the female was removed, male-only test rat USVs were recorded for 90 sec after.

Offline acoustic analysis of USVs was performed with using SASLab Pro (Avisoft, Germany). Individual spectrograms were built from each Avisoft-generated waveform with: Fast Fourier Transform (FFT) of 512 points, frame size of 100%, flat top window, and temporal resolution set to display 75% overlap frame set-up. Additionally, a high pass filter eliminated noise below 25-kHz. Calls were slowed down by 25x to listen and categorize the call type as reported by previous publications [26–28]. Experienced raters, masked to experimental conditions, analyzed the average bandwidth frequency (kHz) and average peak frequency (kHz) of each call [26, 29, 30]. Averages were compiled and statistically analyzed per group (see ‘Statistics’ below).

### Tissue processing

**Fig 1:**
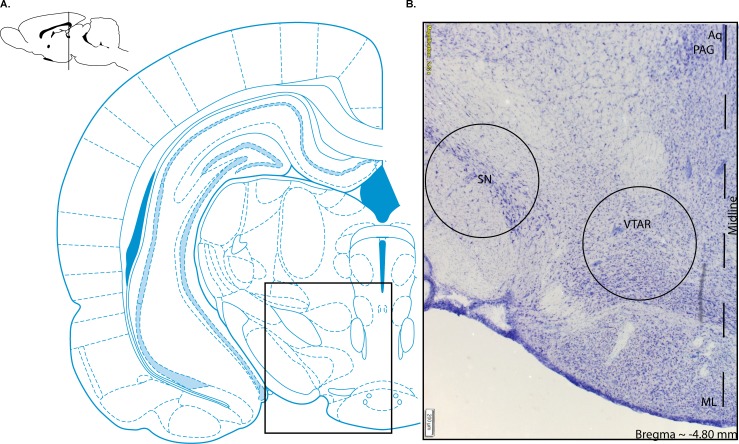
Representative brain schematic. Approximate location and size (2mm) of tissue samples collected for tissue processing (RT qPCR, ELISA, and western blotting). (A) Brain drawing adapted from Paxinos and Watson, 2005. Box shows approximate location of panel (B) representative Nissl stained brain section, 50μm thick section. Photomicrograph at 2.52x magnification. Circular holes were centered within the midbrain and illustrate the approximate location including the ventral tegmental area (approximately Bregma -4.80mm visible at the time of dissection) and adjacent tissue including substantia nigra. Scale bar indicates 200 μm; midline defined by dashed line. Abbreviations: ML = medial mammillary; PAG = periaqueductal gray; SN = substantia nigra; VTAR = Ventral tegmental area (Paxinos and Watson, 2005).

Two days after final testing, all rats were deeply anesthetized with isoflurane and rapidly decapitated. The brains were dissected and immediately frozen and stored at -80° C. Sample order was randomized during processing. Brains were sliced coronally on a cryostat at 250μm thickness at -15°C and mounted on glass slides. A 2 mm in diameter tissue punch was collected from the mesolimbic area including VTA as well as adjacent nuclei (**Fig 1A**; Bregma -4.80mm, approximate) using the Brain Punch Set (FST 18035–02, Foster City, CA, USA) under a dissection microscope over dry ice (**Fig 1B**). Additionally, adjacent 2 mm substantia nigra tissue samples (from the same slide as VTA) were used for RT qPCR and protein analysis as a control region. Anatomically equivalent sections were used from each animal. One set of punches was used for protein, the other set was used for RNA experiments. Tissue samples were transferred to microcentrifuge tubes and stored at -80°C. To maintain consistency, the same pair of researchers extracted all the study samples.

Tissue for protein analysis was homogenized using 200uL of lysis buffer (N-PER Neuronal Protein Extraction Buffer (Thermo Scientific, Rockford, IL, USA) including a cocktail of protease (Sigma Aldrich, St. Louis, MO, USA), phosphatase inhibitors (Sigma Aldrich, St. Louis, MO, USA) and 200mM PMSF (Sigma Aldrich, St. Louis, MO, USA)). Homogenates were incubated on ice for 10 min, centrifuged at 12,000 rpm for 10 min at 4°C to pellet the cell debris. Supernate was collected and stored at -80°C. Total protein concentrations were quantitatively determined using a bicinchoninic acid protein assay (BCA Protein Assay Kit; Thermo Scientific Pierce, Rockford, IL, USA) adopted for microtiter plates for all samples, following the manufacturer’s protocol. The plate was read at 562nm using a BioTek Eon spectrophotometer (BioTek, Winooski, VT, USA) and analyzed with BioTek’s Gen5 v 2.0 data analysis software.

Tissue designated for RNA analysis was homogenized and total RNA was extracted with the Bio-Rad Aurum Total RNA Fatty and Fibrous Tissue Kit (Catalog No. 732–6830; Bio-Rd, Hercules, CA, USA) according to manufacturer’s instructions. Total RNA was measured using a Nanodrop system (Thermo Scientific, Wilmington, DE, USA). DNAse treated RNA (100ng/uL per cDNA reaction) was converted into single-stranded cDNA using the Invitrogen SuperScript III First-Strand Synthesis System (Catalog No. 18080–05; Invitrogen, Carlsbad, CA, USA).

### Protein quantification: ELISA

To quantify the levels of dopamine and norepinephrine, normalized total protein dilutions were plated on a 96-well ELISA plate (BA E-5500; 2-CAT (N-D) Research ELISA, Rocky Mountain Diagnostics, Colorado Springs, CO, USA) according to manufacturer’s instructions and as previously described [57]. Each assay included standards, control solutions, and sample aliquots diluted with distilled water. Absorbance for each well was read at 450 nm on an Eon Plate Reader (BioTek, Winooski, VT, USA). All samples were run in duplicate and within a single assay. Data inclusion criteria consisted of protein concentrations above the manufacturer’s specified limit of detection, intraassay coefficients of variance below 25%, as well as a standard curve R2 of at least 0.990. The total protein concentration in each sample duplicate was averaged for each respective assay; averages for each group were statistically analyzed.

### Primer design and verification

NCBI Primer Blast was used to design primers for control housekeeping genes (*Ywaz* and *Gapdh*) [63] and genes of interest (*Th*, *Gad1*, *Gad2*, *Gls*, *Glul*, *MuR)* using the rat (*Rattus norvegius*) genome. Netprimer (PREMIER Biosoft, Palo Alto, CA, USA) was used to examine secondary structure of all primers. Non-template controls were run with each primer pair to check for formation of primer-dimers and non-specific amplification products. All primer runs yielded single peak melt curves (60°C) indicating amplification of single gene products. Sequences are provided in **Table 2**.

**Table 2:** RT qPCR information.

Gene	Gene Abbreviation	Accession Number	Forward Sequence	Reverse Sequence
tyrosine 3-monooxygenase/tryptophan 5-monooxygenase activation protein, zeta	*Ywaz*	NM_013011.3	GGCAGAGCGATACGATGAC	AGACGACCCTCCAAGATGAC
glyceraldehyde-3-phosphate dehydrogenase	*Gapdh*	NM_017008.4	GGATACTGAGAGCAAGAGAGA	TTATGGGGTCTGGGATGGAA
Tyrosine Hydroxylase	*Th*	NM_012740.3	CTTTGACCCAGACACAGCA	TGGATACGAGAGGCATAGTTC
Glutamate decarboxylase 1	*Gad1*	NM_017007.1	GACACTTGAACAGTAGAGACCC	TGTAGGACGCAGGTTGGTAG
Glutamate decarboxylase 2	*Gad2*	NM_012563.1	CCAGGCTCATCGCATTCAC	GCACTCACCAGGAAAGGAAC
Glutaminase	*Gls*	NC_005108.4	CTGAACGAGAAAGTGGAGACC	GGGCAGAAACCGCCATTAG
Glutamate-ammonia ligase	*Glul*	NM_017073.3	GGGAGGAGAATGGTCTGAGG	TGATGTTGGAGGTTTCGTGG
Mu-opioid receptor 1A	*MuR*	NM_001038597.2	CCGTTTCCTGGCACTTCTG	GTATTAGCCGTGGAGGGATG

RT qPCR information. Gene of interest (abbreviation; http://www.ncbi.nlm.nih.gov/gene), NCBI accession number, Net Primer Score (Premier Biosoft), forward and backward primer sequences for each of the genes tested.

### Quantitative real-time PCR (RT qPCR)

Relative gene expression was determined using real-time (RT) qPCR analysis following the MIQE guidelines for quantitative real-time PCR experiments [53]. All samples were prepared in reaction tubes containing the respective sample cDNA, nuclease-free water, forward and reverse primers (5μM concentration, Integrated DNA Technologies, San Jose, CA, USA) and SsoFast EvaGreen Supermix (Bio-Rad, Hercules, CA, USA). On each plate, five standards were run (1:10 serial dilutions, starting at 500ng/μL) with a non-template negative control. Samples and standards were run in triplicate. Plates were read with the BioRad CFX96 Touch Real-Time PCR Detection System (Bio-Rad, Hercules, CA, USA). Each run entailed an initiation step at 95°C for 30 sec, 40 cycles of 95°C for 5 sec, a 30 sec annealing phase at 60°C for each gene, a 20 sec elongation phase at 72°C, and a melt curve from 60–88°C, 0.5 degrees for each 5 sec step. All plates were read following each elongation and melt curve stage. The mean Ct value for each sample, defined as the average cycle number at which each sample triplicate crossed the amplification threshold, were transformed via the Pfaffl Method [43]. Average relative expression was calculated for each group per gene of interest for both the VTA and substantial nigra.

### Western blot and analysis

For a subset of rats, total protein supernatant was mixed with a pre-calculated volume of 2 × Laemmli buffer (Bio-Rad Laboratories, Hercules, CA, USA) with 2-mercaptoethanol. Extracted protein samples (10 μg of total protein) were denatured at 95°C for 5 min, and lysates were resolved on a Criterion Precast Gel (4–20% gradient Tris HCL-polyacrylamide gels,1.0mm, 12 x 2 Well Comb, Bio Rad). Prestained protein standards (Precision Plus Protein Dual Xtra Standards, Bio Rad) were included on gels as molecular mass markers. Samples were subjected to electrophoresis in 10X Tris-buffered saline buffer with glycine (TBS, Bio Rad) for 1:15 h at 125 V then transferred in 10X TBS with glycine (Bio Rad) with 20% methanol for 1.5 h at 100 V onto 0.2-μm nitrocellulose membranes (Bio-Rad). Membranes were blocked with filtered 5% non-fat milk in Tris-buffered saline containing 0.1% Tween-20 (TBS-T) for 1 h at 4°C with constant agitation. Blots were probed with primary antibodies for GABA (anti-GAD65 + GAD67, 1:1000, #ab11070; abcam Cambridge, MA, USA), mu-opioid receptor (anti mu-opioid receptor, 1:1,000, #abcam10275, abcam Cambridge, MA, USA), and loading control (anti-β actin, 1:40,000, Millipore, Billerica, MA, USA) overnight (minimum 16 hr) at 4°C with constant agitation. Following primary antibody incubation, blots were washed then probed with horseradish peroxidase-conjugated anti-rabbit IgG (1:10,000 dilution, Cell Signaling Technology Inc., Danvers, MA, USA) and anti-mouse IgG (1:20,000 dilution, Cell Signaling Technology Inc.)). Blots were washed in TBS-T and enhanced chemiluminescence substrate with Super Signal West Pico (Thermo Scientific, Madison, WI, USA) was used to develop immunoblots using a ChemiDoc-IT2 Imager (UVP, LLC, Cambridge, UK). For GAD, two bands at 65 kDa and 67 kDa were visible. However, because the band at 67 was faint, the optical density for both GAD bands were analyzed together. For the mu-opioid receptor, the band at 50 kDa was analyzed. ImageJ (National Institutes of Health) was used to analyze grayscale band density normalized to β actin internal controls. For immunoblotting, a Gel Analysis method outlined in the ImageJ documentation was used: http://rsb.info.nih.gov/ij/docs/menus/analyze.html#gels.

### Statistics

All statistical analyses were conducted with SigmaPlot 12.5 (Sys-tat Software, Inc., San Jose, CA, USA). Means and standard error of the means for all data can be found in **Table 3**. A one-way analysis of variance (ANOVA) was used to compare average bandwidth, average peak frequency, average dopamine and norepinephrine protein concentrations, average relative expression of all genes, and average optical density for western blotting between groups (WT, control *Pink1*-/-, sham-exercised *Pink1*-/-and vocal-exercised *Pink1*-/-). The Shapiro-Wilk normality test and Brown-Forsythe equal variance test was used to test assumptions of ANOVA. Fisher LSD method was used for post-hoc analysis. Interclass correlation coefficients were used to determine inter and intra-rater reliability on 10% of the vocalization data; all ICC was above 0.85. Discrepancies in sample sizes presented in **Table 1** are reflective of missing samples or samples that fell below the limit of detection within assays. Critical level of significance was set *a priori* at 0.05.

**Table 3:** Means and standard error of the means in the VTA.

Experimental Variable	Mean (SEM) for WT	Mean (SEM) for control *Pink1* -/-	Mean (SEM) for sham-exercised *Pink1*-/-	Mean (SEM) for vocal-exercised *Pink1* -/-
Bandwidth (Hz)	21496.36 (1185.58)	17377.46 (875.94)	20226.20 (1603.442)	22524.43 (1377.92)
Peak frequency (Hz)	55493.78 (911.83)	54832.25 (597.59)	51357.63 (1920.75)	49253.6 (1286.30)
DA protein (pg/ug total protein)	2.49 (0.039)	2.10 (0.06)	2.001 (0.379)	2.25 (0.90)
NE protein (pg/ug total protein)	2.08 (0.23)	2.33 (0.39)	2.33 (0.52_	1.85 (0.34)
*Th* (relative expression)	1.01 (0.23)	1.03 (0.13)	1.41 (0.633)	1.66 (0.80)
*Gad1* (relative expression)	0.934 (0.29)	0.725 (0.32)	3.83 (0.61)	5.59 (2.68)
*Gad2* (relative expression)	0.617 (0.25)	0.93 (0.30)	2.85 (0.49)	5.23 (2.26)
*Gls* (relative expression)	0.76 (0.17)	0.71 (0.09)	1.98 (0.29)	2.91 (1.12)
*Glul* (relative expression)	1.01 (0.19)	0.64 (0.08)	0.48 (0.24)	1.99 (0.62)
*MuR* (relative expression)	0.702 (0.08)	0.723 (0.12)	1.824 (0.109)	2.07 (1.035)
GAD (pixel intensity)	0.21 (0.043)	0.11 (0.04)	0.32 (0.009)	
MuR (pixel intensity)	0.951 (0.14)	0.66 (0.050)	0.56 (0.06)	

The mean for WT, control *Pink1*-/-, sham-exercised *Pink1*-/-, and vocal-exercised *Pink1*-/- groups with the standard error of the mean for the experimental variables (raw data). Catecholamine, GABAergic, and mu-opioid data for VTA.

## Results

### Average ultrasonic vocalization frequency data

**Fig 2:**
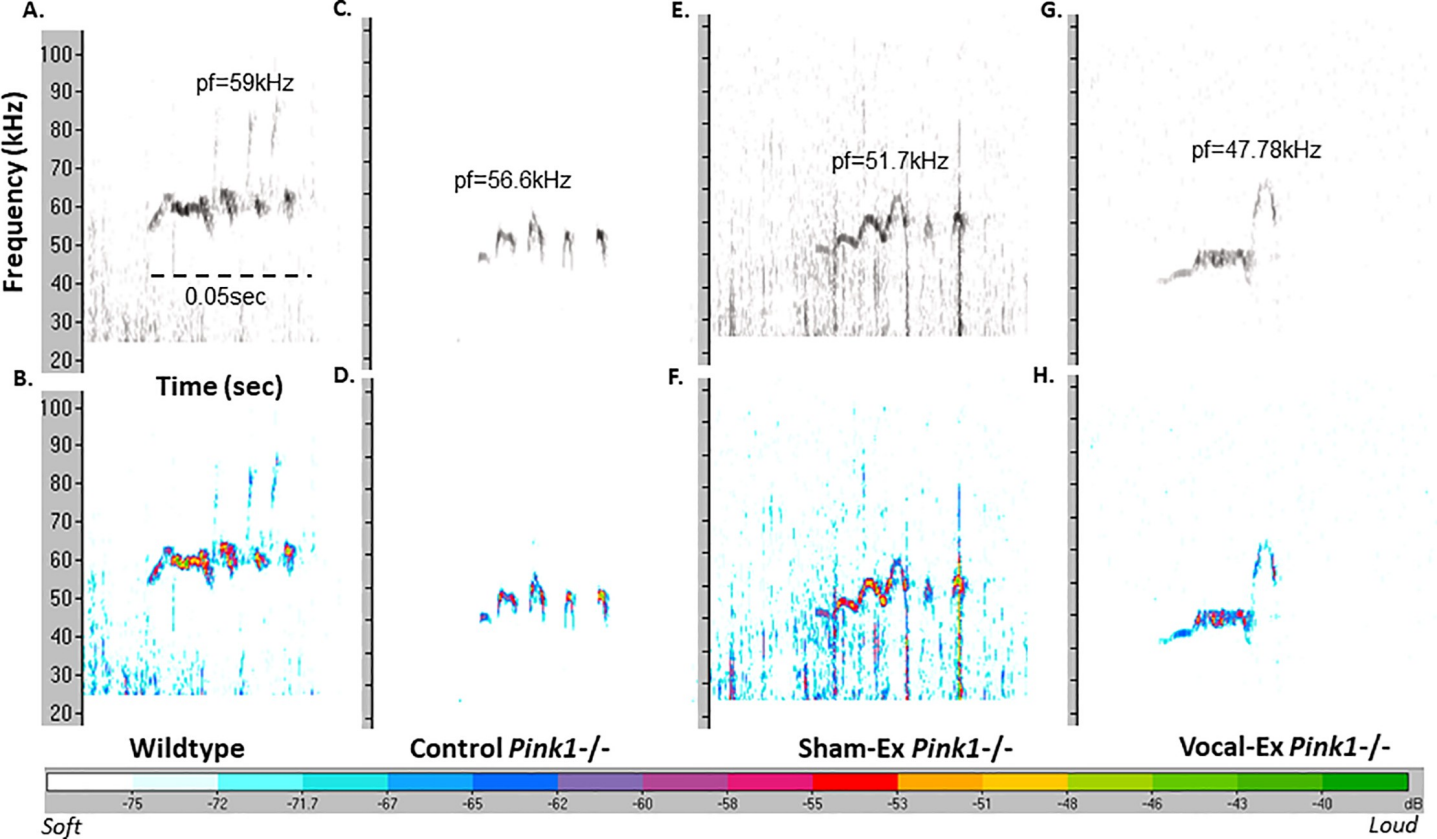
USV spectrogram. Spectrograms (*x* axis: time (sec), *y*-axis: frequency in (kHz)) of complex frequency-modulated USVs in representative samples from (A, B) Wildtype (WT), (C, D) Control *Pink1*-/-, (E, F) Sham-exercised (sham-ex) *Pink1*-/-, and (G, H) Vocal-exercised (vocal-ex) *Pink1*-/- rats at 8 months of age. Within black and white spectrograms: relative intensity (dB, loudness) is encoded by darkness of the signal; darker is louder. Legend for relative intensity for color spectrograms is at the bottom of the Fig Peak frequency (pf) is indicated above each vocalization per group. Scale bar (0.05 sec) is located in panel A.

**Fig 3:**
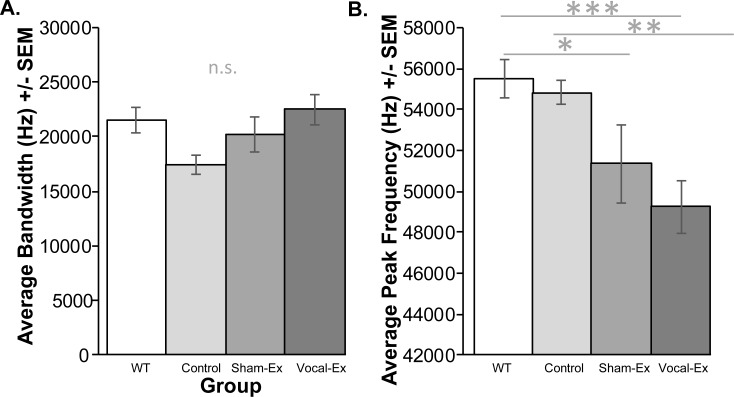
USV frequency data. Average (A) bandwidth (kilohertz (kHz) and (B) peak frequency (kHz) +/- standard error of the mean (SEM). Frequency on the y-axis and group on the x-axis (white bar = wildtype (WT), light gray = control *Pink1*-/-, medium gray = sham-exercised (sham-ex) *Pink1*-/-, dark gray = vocal-exercised (vocal-ex) *Pink1*-/-). Statistical significance between groups indicated by bar and *** p<0.001, **p < 0.01, *p < 0.05. n.s. indicates no significant differences.

Spectrograms of representative frequency-modulated USVs for each group are shown in **Fig 2A–2H**. At 8 mo of age, there were no significant differences in average bandwidth (F(3, 27) = 2.69, *p* = 0.066); **Fig 3A**) among groups (WT, control *Pink1*-/-, sham-exercised *Pink1*-/-, exercised *Pink1*-/-).

There were significant differences between groups for the average peak frequency (F(3, 27) = 6.157, *p* = 0.003); **Fig 3B**). Post-hoc analyses show that there were significant differences between WT and sham-exercised *Pink1*-/- (*p* = 0.02) as well as WT and vocal-exercised *Pink1*-/- rats (p<0.001); both sham- and vocal-exercised rats had decreased peak frequency values compared to WT. There were also significant differences between control *Pink1*-/- and sham/vocal-exercised groups (*p* = 0.005); specifically, all both sham and vocal-exercised rats had significantly reduced peak frequency measures.

### TH mRNA and ELISA catecholamine protein concentrations in the VTA

**Fig 4:**
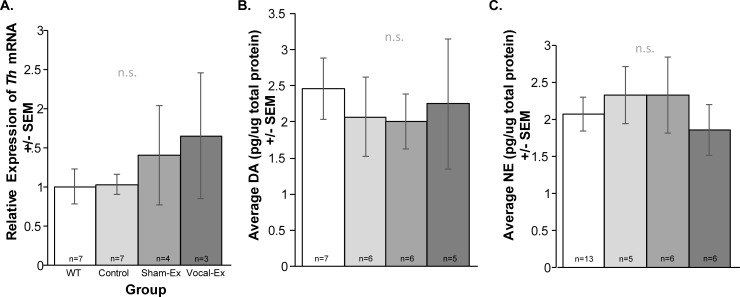
Catecholamine protein concentrations in the ventral tegmental area. Average (A) gene expression for tyrosine hydroxylase (*Th*), (B) dopamine protein concentration and (C) norepinephrine protein concentration +/- standard error of the mean (SEM). Concentration (pg per ug/total protein) on the y-axis and group on the x-axis (white bar = wildtype (WT), light gray = control *Pink1*-/-, medium gray = sham-exercised (sham-ex); dark gray = vocal-exercised (vocal-ex) *Pink1*-/-). n.s. = not significant. Sample size is located inside bars of respective panels.

There were no significant differences between groups (F(3, 17) = 0.0.62, *p* = 0.61; **Fig 4A**) for the relative expression of *Th*. There were no significant differences in dopamine protein concentrations between groups (F(3,20) = 0.0.16, *p* = 0.92; **Fig 4B**). Additionally, there were no significant differences in norepinephrine protein concentrations between groups (F(3,26) = 0.34, *p* = 0.80; **Fig 4C**).

### GABAergic RT qPCR and western blot

**Fig 5:**
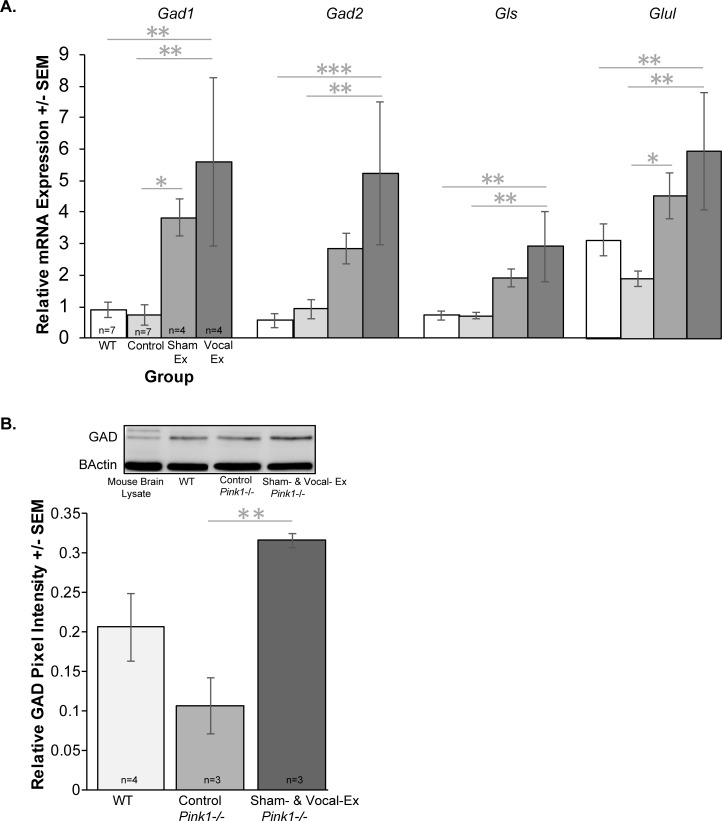
RT qPCR for GABAergic genes in the ventral tegmental area. (A) Average relative mRNA expression values for *Gad1*, *Gad2*, *Gls*, and *Glul* +/- standard error of the mean (SEM). Relative expression on the y-axis and group on the x-axis (white bar = wildtype (WT), light gray = control *Pink1* -/-, medium gray = sham-exercised (sham-ex) *Pink1*-/-, dark gray = vocal-exercised (vocal-ex) *Pink1* -/-). All qPCR data were analyzed with the Pfaffl Method, normalized to reference genes (*Ywaz*, *Gapdh*) expression. (B) Quantification of GAD pixel intensity and representative GAD and β-actin bands on a western blot for each group (WT, control *Pink1*-/-, sham- & vocal-exercised *Pink1*-/-). Statistical significance between groups indicated by bar and *** p<0.001, **p < 0.01, *p < 0.05. Sample size is located inside bars of respective panels.

Similar patterns were observed across GABAergic genes in the VTA (**Fig 5**); there were significant differences between groups for relative mRNA expression for genes implicated in GABA synthesis (**Fig 5A**). For *Gad1* expression in the VTA, there were differences between groups (F(3, 18) = 5.13, *p* = 0.01). Specifically, vocal-exercised *Pink1*-/- rats had upregulated mRNA expression compared to WT (*p* = 0.005) and control *Pink1*-/- (*p* = 0.004). Similarly, sham-exercised *Pink1*-/- had upregulated mRNA expression compared to WT (*p* = 0.061) and control *Pink1*-/- (*p* = 0.047). There were no differences between WT and control *Pink1*-/- rats (*p* = 0.881). Additionally, there were no significant differences between sham-exercised and vocal-exercised (*p* = 0.301).

Similarly, there were significant group differences detected for *Gad2* mRNA expression (F(3, 18) = 5.74, *p* = 0.006); vocal-exercised *Pink1*-/- were significantly increased compared to WT (*p* = 0.001) and control *Pink1*-/- (*p* = 0.003). However, there were no differences between sham-exercised rats and vocal-exercised rats (*p* = 0.12). There were no significant differences between sham-exercised and control (*p* = 0.14) or WT (*p* = 0.73).

Additionally, there were significant differences for *Gls* expression (F(3, 18) = 5.73, *p* = 0.006). Vocal-exercised *Pink1*-/- rats had upregulated expression compared to WT (*p* = 0.002) and control *Pink1*-/- (*p* = 0.002). There were no significant differences between sham- and vocal-exercised rats (*p* = 0.17). There were no differences between sham-exercised and control *Pink1*-/- (*p* = 0.07) and sham-exercised compared to WT (*p* = 0.06). There were no differences between WT and control *Pink1*-/- (*p* = 0.98).

Finally, there were significant differences between *Glul* mRNA expression and group (F(3, 18) = 4.65, *p* = 0.014). There were significant increases in expression in vocal-exercised *Pink1*-/- compared to control *Pink1*-/- rats (*p* = 0.002). Additionally, there were increases in vocal-exercised *Pink1*-/- compared to WT (*p* = 0.0024). While there were no differences between vocal-exercised and sham-exercised (*p* = 0.29), there were differences between sham-exercised and control *Pink1*-/- (*p* = 0.035). There were no differences between WT and sham-exercised (*p* = 0.24) and WT and control *Pink1*-/- (*p* = 0.231).

For western blotting, image analysis software identified a band at 65 and67 kDa specific to the GAD 65+67 protein. There were significant differences between groups (F(2, 9) = 7.604, *p* = 0.018; **Fig 5B**). Specifically, the sham- and vocal-exercised *Pink1*-/- rats had an increased relative pixel intensity compared to control *Pink1-/-* (*p* = 0.006). There were no significant differences between WT and sham- and vocal- exercised *Pink1*-/- (*p* = 0.065), and WT and control *Pink1*-/- rats (*p* = 0.087). There were no differences between any of the groups for the reference protein β-actin (F(2, 4) = 0.028, *p* = 0.97).

### MuR RT qPCR and western blot

**Fig 6:**
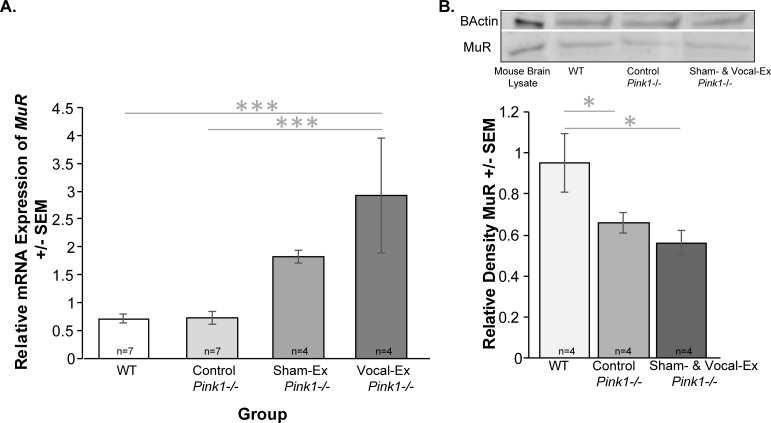
Mu opioid receptor in the ventral tegmental area. (A) relative expression of MuR mRNA expression +/- standard error of the mean (SEM). Relative expression on the y-axis and group on the x-axis (white bar = wildtype (WT), light gray = control *Pink1*-/-, medium gray = sham-exercise (sham-ex) *Pink1*-/-, dark gray = vocal-exercise (vocal-ex) *Pink1*-/-). All qPCR data were analyzed with the Pfaffl Method, normalized to reference genes (*Ywaz*, *Gapdh*) expression. (B) Quantification of MuR protein via western blot and representative MuR bands for each group. Statistical significance between groups indicated by bar and *** p<0.001, **p < 0.01, *p < 0.05. Sample size is located inside bars of respective panels.

Within the VTA there were significant differences between groups for the relative expression of *MuR* (F(3, 17) = 6.58, *p* = 0.004; **Fig 6A**). Vocal exercised *Pink1*-/- rats had increased relative mRNA expression compared to WT (*p*<0.001) and control *Pink1*-/- rats (*p*<0.001). The vocal-exercise group did not differ from the sham-exercise group (*p* = 0.10). Additionally, there was no significant difference between WT and control *Pink1*-/- (*p* = 0.97), sham-exercise and WT (*p* = 0.07), or sham-exercise and control *Pink1*-/- (*p* = 0.07).

Western blot detected a distinct band at 50 kDa that was used for analysis. There was an inverse relationship between *MuR* mRNA expression and densiometric analysis of MuR protein. Specifically, there was a significant difference between groups (F(2, 9) = 4.82, p = 0.038; **Fig 6B**); WT rats had increased MuR densities compared to control *Pink1*-/- rats (*p* = 0.05) and sham & vocal-exercised *Pink1*-/- (*p* = 0.015). There were no significant differences between *Pink1*-/- groups (*p* = 0.48).

### Control RT-qPCR

Reference genes were also compared to confirm no differences between groups. For VTA, there were no differences in the relative expression of *Ywaz* (F(3,18) = 0.77, *p* = 0.53) or *Gapdh* (F(3, 18) = 0.1.003, *p* = 0.41) between groups.

**Fig 7:**
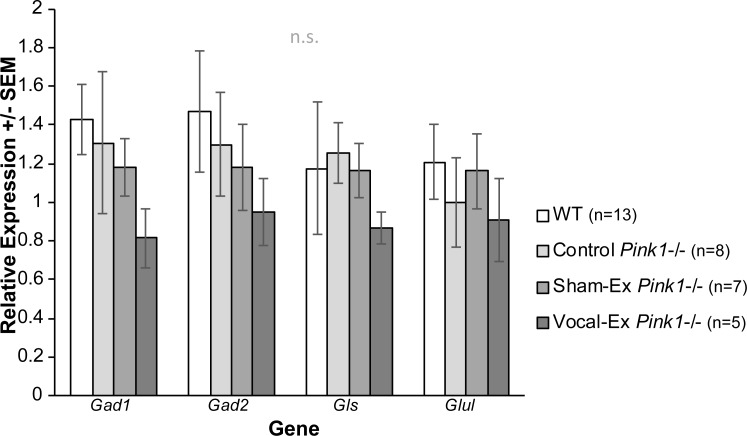
GABAergic gene expression in the substantia nigra. (A) Average relative mRNA expression values for *Gad1*, *Gad2*, *Gls*, and *Glul* +/- standard error of the mean (SEM). Relative expression on the y-axis and group on the x-axis (white bar = wildtype (WT), light gray = control *Pink1* -/-, medium gray = sham-exercised (sham-ex) *Pink1*-/-, dark gray = vocal-exercised (vocal-ex) *Pink1* -/-). All qPCR data were analyzed with the Pfaffl Method, normalized to reference genes (*Ywaz*, *Gapdh*) expression. n.s. indicates no significant findings. Sample size is located inside bars of respective panels.

To demonstrate regional specificity with the findings, adjacent tissue containing the substantia nigra was used as a control. Mean (SEM) for data are found in **Table 4**. There were no significant differences between groups for reference genes, *Th*, GABAergic genes, and *MuR* expression (*p*>0.05 for all relationships; **Fig 7**).

**Table 4:** Means and standard error of the means in the SN.

Experimental Variable	Mean (SEM) for WT	Mean (SEM) for control *Pink1* -/-	Mean (SEM) for sham-exercised *Pink1*-/-	Mean (SEM) for vocal-exercised *Pink1* -/-
*Gad1* (relative expression)	1.43 (0.18)	1.31 (0.31)	1.18 (0.35)	0.82 (0.20)
*Gad2* (relative expression)	1.47 (0.36)	1.30 (0.27)	1.18 (0.16)	0.95 (0.23)
*Gls* (relative expression)	1.18 (0.15)	1.25 (0.22)	1.17 (0.14)	0.87 (0.19)
*Glul* (relative expression)	1.21 (0.15)	1.0 (0.17	1.16 (0.082)	0.91 (0.21)

The mean (SEM) for WT, control *Pink1*-/-, sham-exercised *Pink1*-/-, and vocal-exercised *Pink1*-/- groups with the standard error of the mean for the experimental variables (raw data).

## Discussion

Early-stage PD, prior to clinical deficits, is hypothesized to manifest in brainstem regions and non-dopaminergic systems are often dysregulated. At 8 mo, the male *Pink1-/-* rat model of PD exhibits the onset of detectable limb motor and cranial sensorimotor (vocal) deficits as well as non-dopaminergic pathology including reduced norepinephrine concentrations in the locus coeruleus as well as alterations in gene expression, and aggregated alpha-synuclein in the periaqueductal gray and other brainstem regions [52–54, 57, 58, 64]. In this study, we examined the neurochemistry of the mesolimbic VTA with the specific hypothesis that *Pink1-/-* rats that underwent a chronic, daily vocalization exercise treatment (for 6 mo total) would demonstrate differential expression of catecholamines, GABA, and mu-opioid receptor in the VTA as compared to age-matched WT and *Pink1-/-* controls (both controls were non-exercised). A secondary hypothesis of this work tested the changes due to rewarding behavioral intervention and increased social contact (sham-exercised rats). In general, the vocal-exercised rats displayed the greatest change, although not statistically different from the sham-exercised rats, in the peak frequency acoustic measure of USVs. However, while the vocal-exercise group did not differ from the sham-exercised group in terms of vocal behavior, both groups did exhibit higher GABAergic expression levels compared to both control WT and control/non-exercised *Pink1*-/- rats. Vocalization exercise does appear to show positive effect on the average frequency range of the USV when compared to *Pink1* (unexercised) controls suggesting a positive vocal intervention in this PD model. There were no changes in catecholamine concentrations among any of the groups, similar to an early disease stage model, and there was an inverse relationship between mu-opioid receptor gene and protein expression. In general, these results support the assertion that the modulation of the VTA is involved in USV peak frequency modulation in the *Pink1*-/- rat undergoing vocal-exercise experimentation and that the rewarding nature of the experimental protocol may influence and modulate VTA neurotransmitters.

Vocal deficits in PD do not respond positively to dopamine replacement or high frequency deep brain stimulation (both focused on the primary disease pathology of the substantial (>80%) dopamine loss in the nigra). The currently hypothesis is that pathology outside of the nigro-striatal pathway may lead to these earlier deficits (up to a decade prior to a clinical diagnosis). To that end, exercise-based voice training improves vocal quality in humans [12, 14], in PD rodent models including the 6-OHDA model [61], and the early-onset genetic model of PD, the *Pink1*-/- rat [58]. Here, the behavioral data suggest that the vocalization paradigm that is used in the laboratory to chronically (daily over several months) stimulate vocalizations, as a parallel to human therapy, does have a positive effect on the frequency range of vocalizations when compared to normal WT controls. Further, the sham-exercise arm of the study suggests that part of the modulation of behavior may be related to reward. *Pink1*-/- rats that undergo this treatment have increased frequency range (bandwidth) compared to pair-housed *Pink1*-/- control rats (although not statistically significant in this study). Importantly, the means of the vocal-exercised rats are identical to WT (and slightly increased compared to the sham-exercised group). Our previous studies have shown that for non-exercised *Pink1*-/- rats at 8 mo of age, the bandwidth of the 50-kHz vocalizations is significantly reduced [52] which is similar to the finding in **Fig 3A**. The current study is the first to suggest that chronic behavioral modification via vocal exercise does affect bandwidth akin to the results of human vocal therapy (i.e. LSVT Loud). However, within the sham- and vocal-exercised rats, the average peak frequency measure is significantly decreased compared to both the WT and *Pink1*-/- control rats. Previous work has suggested that the peak frequency measure, the frequency at which the loudness part of the call is emitted, is an important component of these prosocial calls [44]. This specific biological measure may play an important role in establishing social proximity and regulating mating behavior; thus, a rat that has daily access to females (as both sham- and vocal-exercised rats did) during the vocalization training may, over time, exhibit a reduction in peak frequency.

The mesolimbic reward system is biologically necessary for associating stimuli with motivationally-relevant outcomes, and VTA activity stimulates behavioral responses to reward as well as the stimuli that predict the reward. During conditioning VTA neurons are initially activated by reward and then over time, the activity shifts to fire at the onset of reward stimuli [65]. The experimental procedure used to elicit USVs in the laboratory is both a social and reward-based task. First, in order to elicit an increased number of USVs the male is allowed to interact with a female rat in estrous. The anticipation of a social reward has been shown to influence USVs [36]. Neuromodulators, including oxytocin and vasopressin, in the VTA activate this region and the reward pathway during social encounters [66, 67]. Second, in order to maintain daily compliance with the vocalization task, the experimental rats are water-restricted; thus, they are highly motivated to produce USVs for a water reward, thus directly activating rewarding circuitry. We would expect both of these variables to contribute to gene and protein expression changes within the VTA, and also influence the projections from the VTA to other brain regions within the mesocorticolimbic circuit. Rats were not water restricted before euthanasia. However, GABA neurons also increase their firing rates during cues that predict reward, in this case it could be the transportation from the animal facility to the experimental room and the experimental procedures/student experimenters that influence activity of this region [68]. Methodologically, in this study rats were transported from the lab animal housing facility to the observation room, which consisted of loading the cages on a transport cart which was wheeled throughout the building to the lab (approximately 5 min) on each test day. The interplay between GABA and DA neurons controls the initiation and termination of reward-related behaviors. Interestingly, there were no changes in *Th* expression and catecholamine concentrations within the VTA. However, future work should look for changes in neurotransmitters and other neuromodulators in projection regions including the nucleus accumbens.

The data that show no significant difference in catecholamines in this study is also consistent with the ‘preclinical’ timepoint of this specific PD model. Our past data has shown no significant loss of dopamine in the substantia nigra and the striatum [52, 57]. Similar to human patients, administration of Levodopa, does not dramatically improve vocal behavior in this model [57, 69]; a limited response to dopamine replacement may be due to the activity of dopamine receptors, synaptic plasticity, or metabolism of the neurotransmitter prior to activating brain regions. Alternatively, the noradrenergic system influences vocalizations [70, 71], and is degenerated in early-PD [26, 72–74]. Modulation of this system may be a critical target for future studies. Overall, these results are consistent with a Braak-staging hypothesis [75, 76], and rostrally-ascending early-stage PD disease model [77]. While the VTA is a dopamine-rich and involved in vocalization frequency modulation, there are several other modulators within this nucleus that may influence vocalizations, including GABA, as discussed below.

A substantial finding in this series of experiments, is the significant difference in GABAergic gene and protein expression in the VTA of the sham- and vocal-exercised *Pink1*-/- rats. All GABA genes (*Gad1*, *Gad2*, *Gls*, *Glul*) showed the same pattern of expression across groups, where the treated *Pink1*-/- rats had significantly (approximately 4 times for sham-exercised; 5 fold for vocal-exercised) upregulated gene expression, a finding that was confirmed with a follow-up western blot for GAD in the VTA of the same rats. We acknowledged that upregulation of GABA may be due to the experimental paradigm as there was no quantitative difference between the WT and the control *Pink1*-/- groups. This work did not look at specific GABA receptor expression; however, previous work in mouse pups has shown that USVs are reduced with benzodiazepine administration (GABA receptor modulation). Several compounds that influence the GABAergic system have been found to modulate the number and acoustic parameters of USVs, including methylphenidate and amphetamine. Additionally, the plasticity of the inhibitory synapses, receptor regulation, and neurotransmitter projections may contribute to behavioral vocalization adaptions observed here; however, this was not directly tested. Additionally, the observed increase in GABA inhibition may be influencing the peak frequency acoustic parameter and reflect the significant decrease in this measure. The interplay between GABA and dopamine in the VTA may serve as a potential site for pharmacological therapy in the mid-to-late stages of the disease when GABA and Ca^2+^ decline significantly and may contribute to the damage of dopamine neurons and accumulation of alpha synuclein protein [27]. Together, the current data support the working hypothesis that an increase in GABA in the VTA may be modulating vocal peak frequency in the rat when undergoing experimental manipulation, and the impact of the increase of GABA in this region on the dopaminergic projection neurons is an area for future study.

Opioids are essential for the anticipation of rewarding, affective, and sensorimotor-related activities including vocal behaviors, and neurotransmission consequently affects downstream behavioral responses [78]. The data from this experiment show that sham-exercised *Pink1-/-* rats have significant, 2 times, upregulation of mu-opioid receptor expression and vocal-exercised rats had a 3-fold increase; however, all *Pink1-/-* rats, regardless of vocalizing treatment, exhibit decreases in mu-opioid receptor protein content compared to WT controls. We acknowledge that this difference could be due to the time course of the study (euthanasia two days after behavioral conditioning was terminated). For example, within the VTA, opioids have been implicated in a variety of rat behavior including juvenile play, social approach, and attachments. Moreover, there is a strong association between opioids and vocalization production. Injections of mu-opiate agonists directly into the VTA produces increases in 50-KHz calls [50]. There is also evidence that suggests that repeated social stress upregulates VTA mu-opioid receptors [79]. Optogenetic studies show that VTA neurons respond to reward-predicting cues as well as aversive stimuli [68]. It is possible that gene expression is increased in the VTA due to behavioral treatment differences; rats that were rewarded for vocalizing with a water treatment would likely have increased gene expression as demonstrated here. However, an interesting finding is the reduced mu-opioid receptor protein content in the *Pink1* genotype compared to WT. There is evidence to suggest that opioid receptors are impacted in early-stage PD; for example, sensory systems are altered [80] and anhedonia, apathy and dysphoria are non-motor symptom of the disease [81]. Differences in opioid receptors, including kappa and dynorphin, and their respective precursors and ligands should be further investigated in the *Pink1*-/- model.

Vocalization is a complex sensorimotor task that is regulated by neural substrates and modulators in multiple brain regions. In the early stages of PD, voice is negatively impacted yet can be rescued by clinical voice exercise therapy. The mechanisms of how this therapy modulates the brain is unknown. This study is the first to suggest that the vocalization paradigm changes mesolimbic modulation activity that influences the PD rat vocalization (peak frequency), and further suggests that social and rewarding behaviors could change vocal behavior and offer a target for treatment.
